# Evaluation of the Presence of Porphyromonas gingivalis and Its Correlation With Signs and Symptoms in Endodontic-Periodontal Lesions: A Cross-Sectional Study

**DOI:** 10.7759/cureus.91075

**Published:** 2025-08-26

**Authors:** Drisya Soman, Moksha Nayak, Mahesh CM, Rahul Radhakrishnan, Praveena G, Nisha B Kurup

**Affiliations:** 1 Department of Conservative Dentistry and Endodontics, Azeezia College of Dental Sciences and Research, Kollam, IND; 2 Department of Conservative Dentistry and Endodontics, KVG Dental College and Hospital, Sullia, IND

**Keywords:** culture, endodontic-periodontal lesions, microorganism, pain, periapical lesion index

## Abstract

Introduction: Endodontic-periodontal lesions are infections that simultaneously affect the pulp and periodontal tissues, often leading to complex clinical presentations. *Porphyromonas gingivalis*, a key periodontal pathogen, has also been identified in root canal infections, suggesting a potential role in the severity and progression of endodontic-periodontal lesions. Its virulence factors contribute to tissue destruction, inflammation, and immune modulation. However, data correlating its presence with specific clinical signs and symptoms in endodontic-periodontal lesions remain limited. This study aims to evaluate the presence of *Porphyromonas gingivalis* and its association with clinical and radiographic parameters in endodontic-periodontal lesions.

Methodology: Clinical and radiographic findings considered for the diagnosis of endodontic-periodontal lesions were recorded from 32 patients, following which root canal samples were obtained and transported for identification of *P. gingivalis* by culture. Fifteen root canal samples positive for *P. gingivalis *species confirmed by culture were considered for the study. The results were assigned, and statistical analysis was done using the chi-square test, Fisher's exact test, and the Mann-Whitney U test.

Results: *P. gingivalis *was detected in 46.9% (15/32) of endodontic-periodontal lesions. Among *P. gingivalis*-positive cases, the sinus tract was present in 66.7% (2/3), swelling in 71.4% (5/7), tenderness on percussion in 46.9% (15/32), and discoloration in 60% (3/5). The organism was more frequent in symptomatic cases (65.5%; 15/23), with statistically significant associations observed for pain (*P* < 0.05) and furcation involvement (88.9%; 8/9; *P* < 0.05). On periodontal evaluation, *P. gingivalis*-positive patients had greater mean pocket depth (5.33 mm) and attachment loss (4.93 mm) compared to negative patients (*P* < 0.05). Higher PAI scores (≥4) were recorded in 80% (12/15) of positive cases, with score 5 in 53% (8/15), score 4 in 26.7% (4/15), and score 3 in 20% (3/15). Periapical lesion size between 3 and 5 mm was observed in 60% (9/15) of positive cases, while 40% (6/15) had lesions >5 mm, showing a significant association with *P. gingivalis* presence (*P* = 0.001).

Conclusion: *P. gingivalis* is strongly associated with clinical signs and symptoms and its detection frequency positively correlates with the severity of endodontic-periodontal lesions.

## Introduction

The pulp and periodontium are closely interconnected, and their health, function, and disease processes are often interdependent [1]. Endodontic-periodontal lesions typically emerge due to shared anatomical and vascular pathways such as the apical foramen, lateral and accessory canals, interradicular canals, and dentinal tubules that link the pulp and the surrounding periodontium [2]. These lesions can result from infections of either origin and are associated with clinical signs like pulp necrosis, increased pocket depths, localized inflammation or swelling, bleeding on probing, pus discharge, sinus tract formation, percussion sensitivity, tooth mobility, bone loss, and pain [3]. Studies investigating the microbiota associated with both primary and secondary endodontic-periodontal lesions have identified a diverse range of microorganisms, including obligate anaerobes, facultative anaerobes, aerobes, and viruses [4-6]. Among these, *Porphyromonas gingivalis*,* *a gram-negative anaerobe from the red complex group, is frequently identified using anaerobic culture methods and has been reported in approximately 61% of endodontic samples from such lesions. Its virulence factors, including gingipains and lipopolysaccharides (LPS), contribute to tissue destruction, inflammation, and immune evasion, which may be associated with more severe clinical signs and radiographic findings [6]. This highlights the importance of examining the relationship between *P. gingivalis* and the clinical manifestations of endodontic-periodontal infections. Hence, the aim of the present study was to evaluate the presence of *P. gingivalis *and its correlation with clinical signs and symptoms in endodontic-periodontal lesions.

## Materials and methods

Source of the data

This observational cross-sectional study adhered to the Strengthening the Reporting of Observational Studies in Epidemiology guidelines [7]. Root canal samples from teeth with endodontic-periodontal lesions requiring endodontic treatment were collected from patients aged 30-70 years referred to the Department of Conservative Dentistry and Endodontics, KVG Dental College and Hospital, Sullia, India. The study protocol was approved by the Institutional Ethical Committee (ACA/DCD/SYN/KVGDC-S/PG/2015-16), and the study was conducted from March 2017 to September 2017. Informed written consent was obtained from all participants after explaining the study objectives and procedures.

The exclusion criteria comprised patients with systemic conditions known to affect periodontal health (such as uncontrolled diabetes and immunocompromised states), those who had undergone antibiotic or periodontal therapy within the past three months, pregnant or lactating women, individuals with a history of tobacco use, teeth presenting with root fractures or calcified canals, and those that had previously undergone endodontic treatment.

To minimize potential confounding factors, only systemically healthy, nonsmoking patients with untreated endodontic-periodontal lesions were included. Standardized protocols were followed for sample collection and analysis.

All clinical and radiographic examinations, diagnoses, and patient recruitment were carried out by a single trained examiner to minimize interexaminer variability. The examiner was calibrated before the study, and reliability was verified by reexamining a subset of patients after two weeks, with findings consistent with the initial recordings.

Sample size estimation

**Figure 1:**
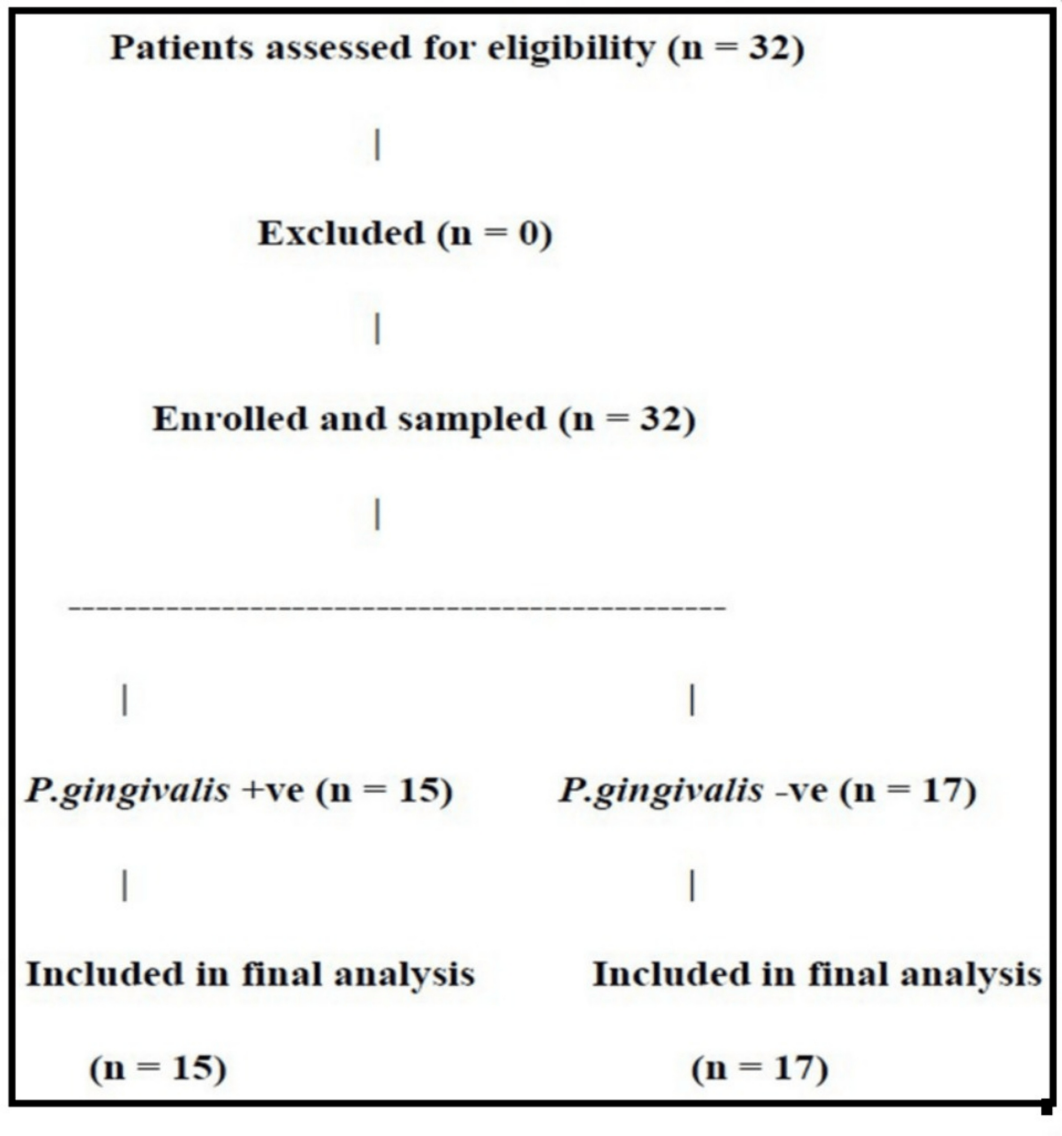
Patient recruitment flowchart as per STROBE guidelines STROBE: Strengthening the Reporting of Observational Studies in Epidemiology

Assuming that the minimum difference of parameters between any two groups is 2 mm, the sample size estimation was made (Figure 1). The expected difference between the parameters of any two groups (d) is 2 mm, the standard deviation (σ) is 1.5, the power of study is (1 - β) = 90%, where β = 10%, and α error is 5%, and n is 12.288 = 15, which is calculated using the following formula:

$n = \frac{2 \times \sigma^2 \times \left(Z_{1-\alpha} + Z_{1-\beta} \right)^2}{d^2}$.

Diagnosis of endodontic-periodontal lesions

Lesion Classification

Patients with combined endodontic-periodontal lesions were included in the study, diagnosed according to Simon’s classification (1972) [1]. No separate categorization into primary endodontic, primary periodontal, or secondary lesions was applied; inclusion was based on the presence of concurrent pulpal and periodontal involvement confirmed clinically and radiographically.

**Figure 2:**
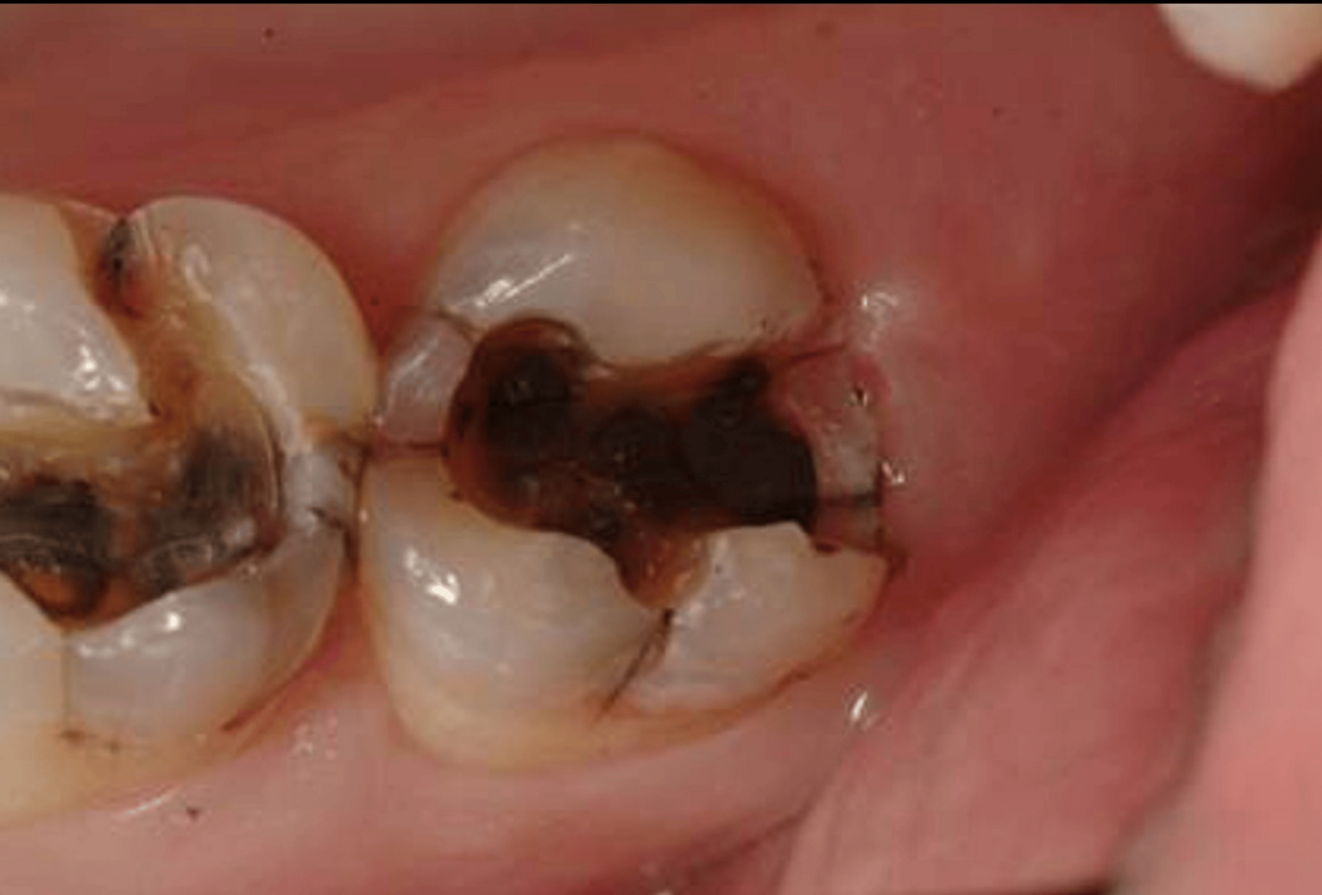
Signs and symptoms of patients positive to P. gingivalis with endodontic-periodontal lesions showing caries

**Figure 3:**
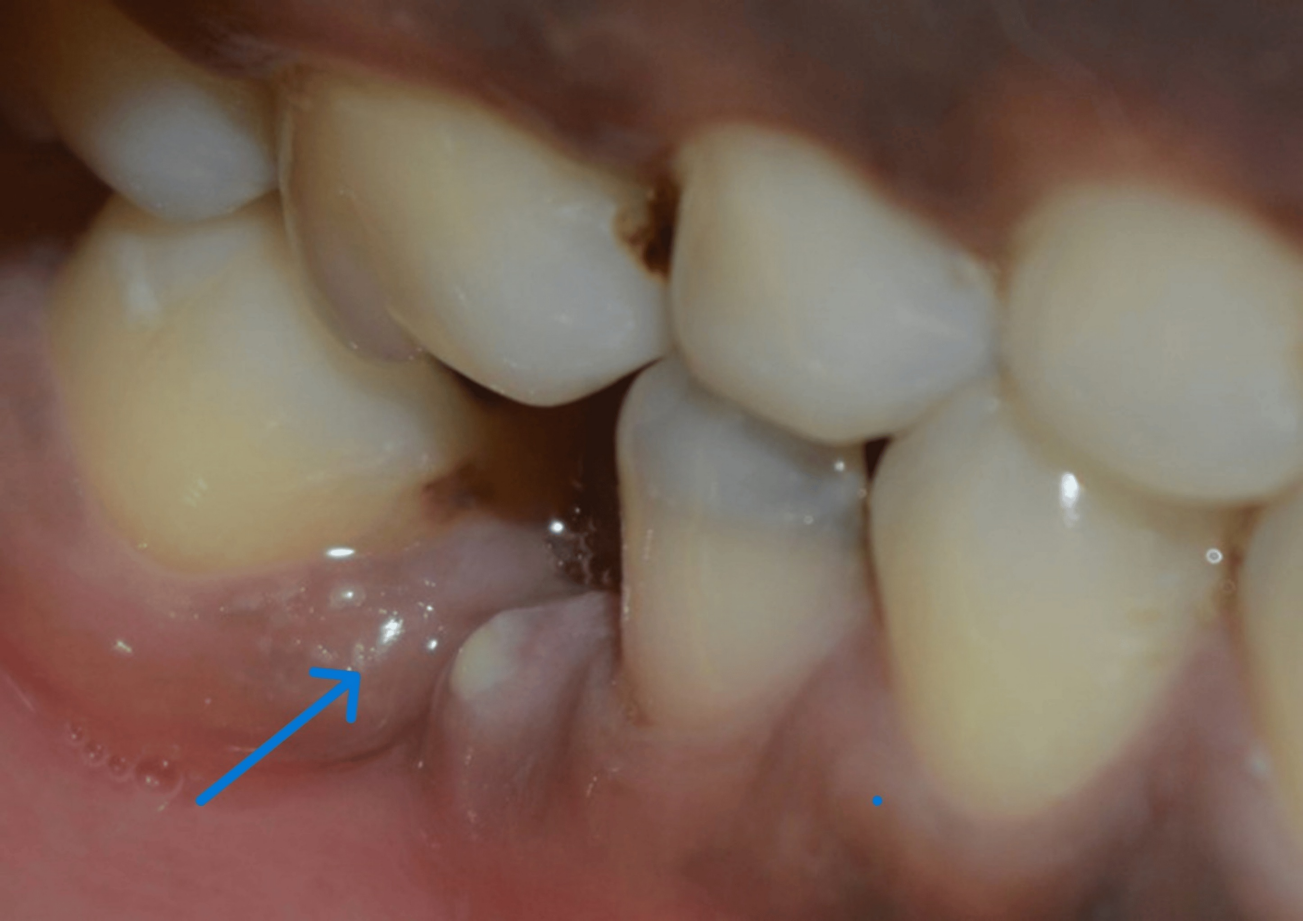
Signs and symptoms of patients positive to P. gingivalis with endodontic-periodontal lesions showing swelling

**Figure 4:**
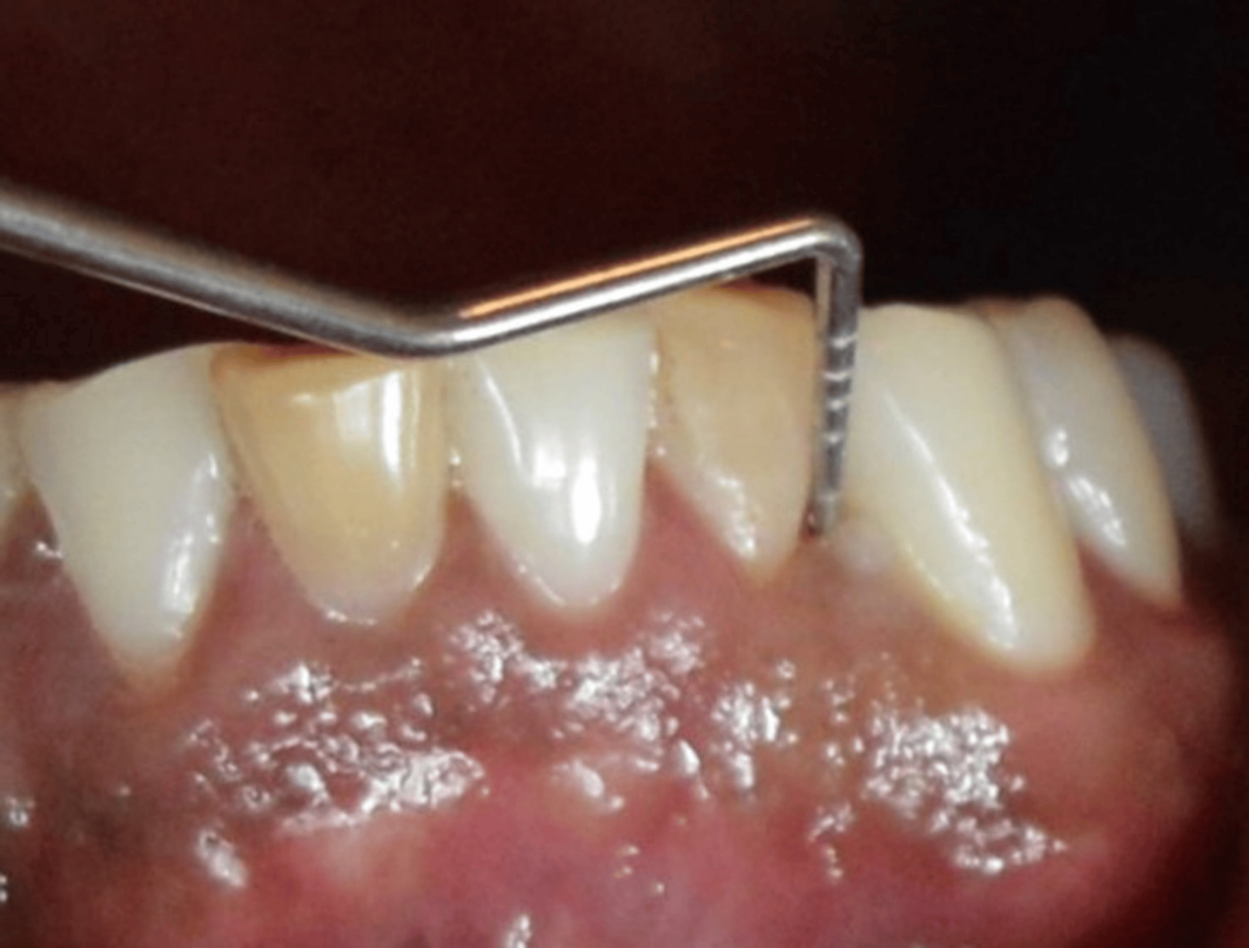
Signs and symptoms of patients positive to P. gingivalis with endodontic-periodontal lesions showing pocket depth

**Figure 5:**
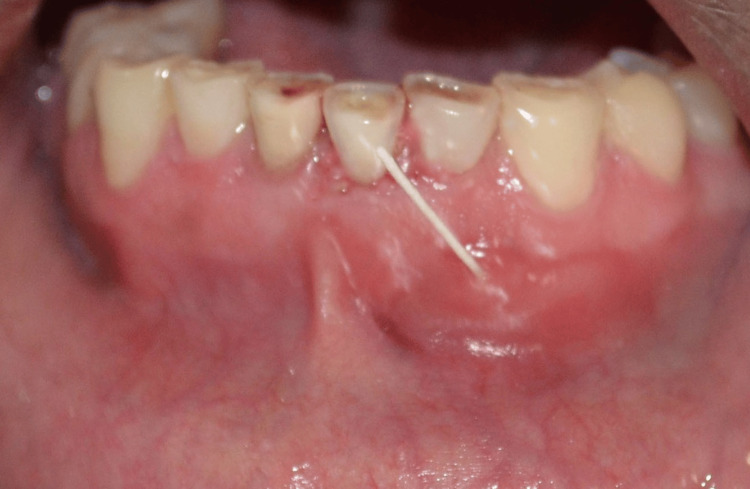
Signs and symptoms of patients positive to P. gingivalis with endodontic-periodontal lesions showing sinus tract opening

**Figure 6:**
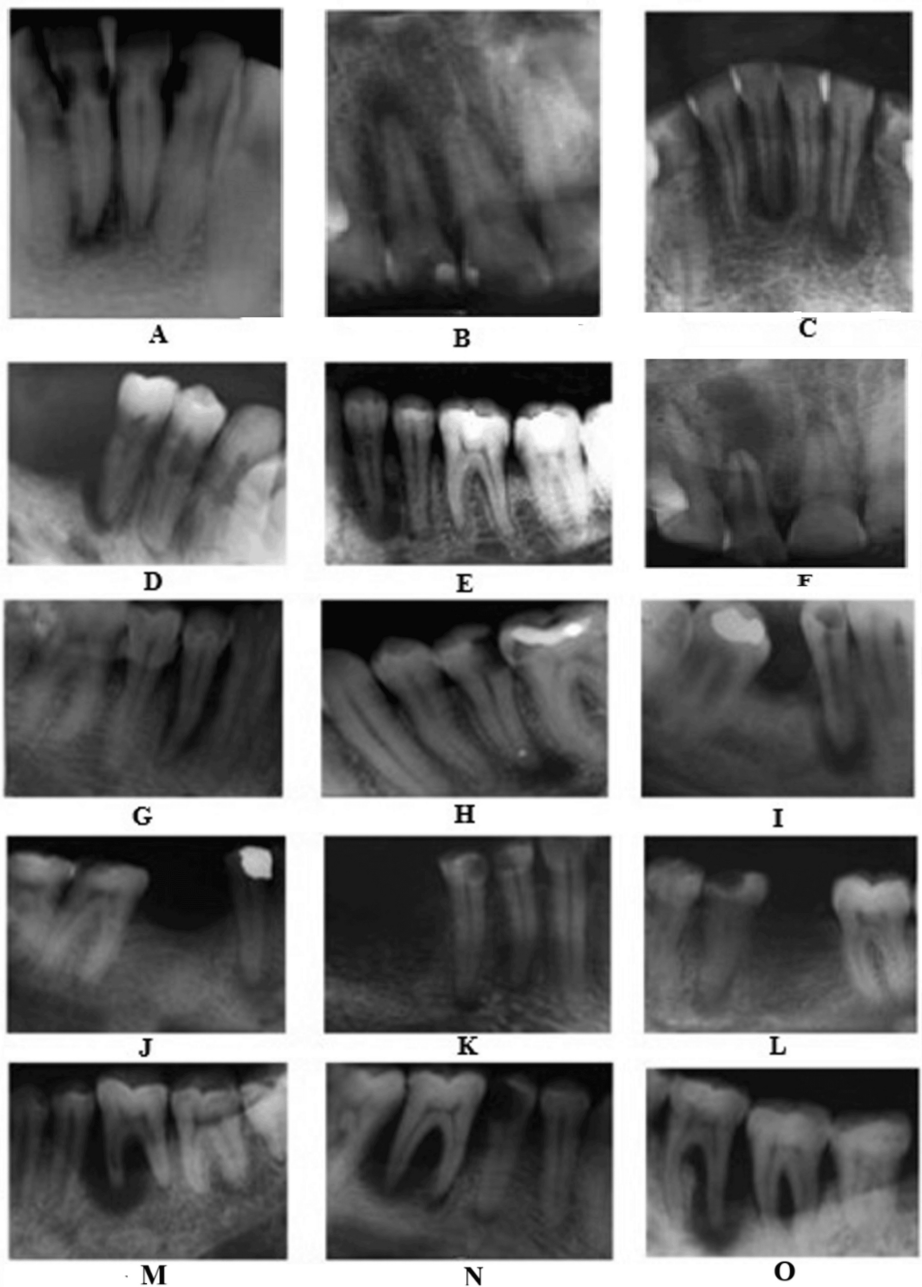
Periapical radiographs of patients positive to P. gingivalis with endodontic-periodontal lesions (A) Periapical lesion 31, 41. (B) Periapical lesion 11, 12. (C) Periapical lesion 41, 32. (D) Periapical lesion and bone loss 45. (E) Periapical lesion 34 and periodontal space widening with periapical changes 35. (F) Large periapical lesion 12. (G) Periapical lesion and vertical bone loss 44. (H) Caries and periapical lesion 35. (I) Periapical lesion and bone loss 45. (J) Coronal radiopacity and periapical lesion 44. (K) Caries and periapical lesions 44, 45. (L) Caries and periapical lesion 35. (M) Large periapical lesion with severe bone loss 36. (N) Large periapical lesion with severe bone loss 46. (O) Large periapical lesion with severe bone loss 36, 37.

Case history and clinical examination of all the subjects were recorded. Clinical and radiographic findings considered for the diagnosis of endodontic-periodontal lesions included nonvital tooth, irreversible pulpitis with chronic apical periodontitis, periodontal probing depth >4 mm, clinical attachment loss >4 mm, bone loss, periapical index (PAI) score of 3 or >3, lesion size of greater than 3 mm, furcation involvement of class II or more, mobility, presence of inflammation and bleeding on probing (Figures 2-6).

Clinical Evaluation of Nonvital Teeth With Endodontic-Periodontal Lesion

Subjects were evaluated for pulp sensitivity tests, which included thermal and electric tests. The cold test was performed by placing ice sticks on the labial surface of the tested teeth. The heat test involved a heated gutta-percha cone placed on the middle third of the tooth.

The electrical test was performed using the Parkell Pulp Vitality Tester (Parkell Inc., Edgewood, NY). The electrical test was performed with an electrode gel used as the contact substance between the tooth tested and the probe tip. A gentle pulse stimulus of 1-10 mA was then generated. Teeth that showed no response to the sensitivity tests were included in the study [8].

Pocket Depth

The periodontal probe (PCP 15; Hu-Friedy, Chicago, IL) was inserted parallel to the vertical axis of the tooth and "walked" circumferentially clockwise around each surface of the tooth to detect the area of deepest penetration. Subjects with a periodontal probing depth of >4 mm were selected for the study [9].

Clinical Attachment Loss

Clinical attachment loss is the distance from the gingival margin to the base of the pocket minus the distance from the gingival margin to the cementoenamel junction. Clinical attachment loss of >4 mm was included in the study [10].

Bleeding on Probing

After measuring the probing depth, the corresponding sites (buccal and mesiobuccal) were inspected for the presence or absence of bleeding and noted in an evaluation chart by probing gently along the wall of soft tissue of the gingival sulcus. If the percentage of sites with bleeding on probing for each person is less than 30% of all probed sites, it was defined as local bleeding only. A percentage of 30% or higher of sites was considered general bleeding on probing [11].

Tooth Mobility

All teeth were examined to determine mobility. The tooth was moved between an instrument handle and an index finger in a buccolingual direction (and mesiodistal direction when no adjacent tooth is present). The amplitude of movement of the crown tip from its most extreme lingual (or distal) position was considered. Visible mobility was scored as follows (Miller’s Index): score 1: mobility up to 1 mm; score 2: mobility of 1-2 mm; score 3: mobility over 2 mm; and/or rotation or depression [12].

Furcation Involvement

Furcation involvement was assessed using Glickman's classification with a Nabers probe, wherein class I denotes incipient involvement, class II represents a cul-de-sac defect with possible vertical bone loss, class III indicates through-and-through involvement, and class IV signifies through-and-through involvement without soft tissue coverage, allowing complete visualization. Cases with class II or higher involvement were included in the study [13].

Preoperative assessment of the periapical status was assessed based on the criteria by Ørstavik et al. [14] using the PAI, which utilizes a radiographic scoring system from 1 to 5 based on periapical bone changes. A score of 1 indicates a normal periapical structure, 2 suggests minor bone alterations not necessarily pathognomonic, 3 represents changes with some loss of mineralization typical of apical periodontitis, 4 denotes a well-defined radiolucent area suggesting established apical periodontitis, and 5 reflects severe bone changes with exacerbating features. The PAI score of 3 or >3 was included in the study.

Microbial sampling of the canals

Isolation and Tooth Decontamination

All treatment and sampling were performed in a single visit by the same endodontic specialist under strict aseptic conditions. Teeth were cleaned with pumice, isolated with a rubber dam, and disinfected according to Möller’s protocol [15] using 30% hydrogen peroxide (H₂O₂) and 2.5% sodium hypochlorite (NaOCl), followed by neutralization with 5% sterile sodium thiosulfate.

Endodontic Access and Disinfection

Standard access cavities were prepared with sterile burs, and defective restorations and caries were completely removed to prevent contamination. The tooth and rubber dam were redisinfected with 30% H₂O₂ and 2.5% NaOCl, then neutralized with 5% sodium thiosulfate.

Verification of Disinfection

Two sterile saline-moistened cotton pellets were used to swab the access cavity and tooth surface. Pellets were transferred to vials containing 1 mL reduced transport fluid (RTF) for microbiological analysis. Samples showing bacterial growth were excluded from the study.

Specimen sampling

**Figure 7:**
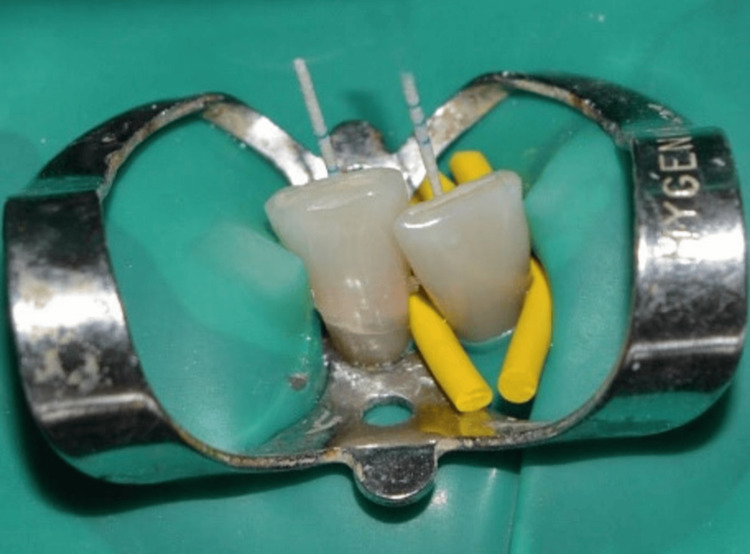
Root canal sampling obtained using absorbent points

After access cavity preparation, root canal samples were collected as per Moller’s criteria [15] using sterile absorbent points (Figure 7). The root canal samples placed in reduced transport media (RTF) were transported for identification of *P. gingivalis *by culture.

Identification of bacteria

**Figure 8:**
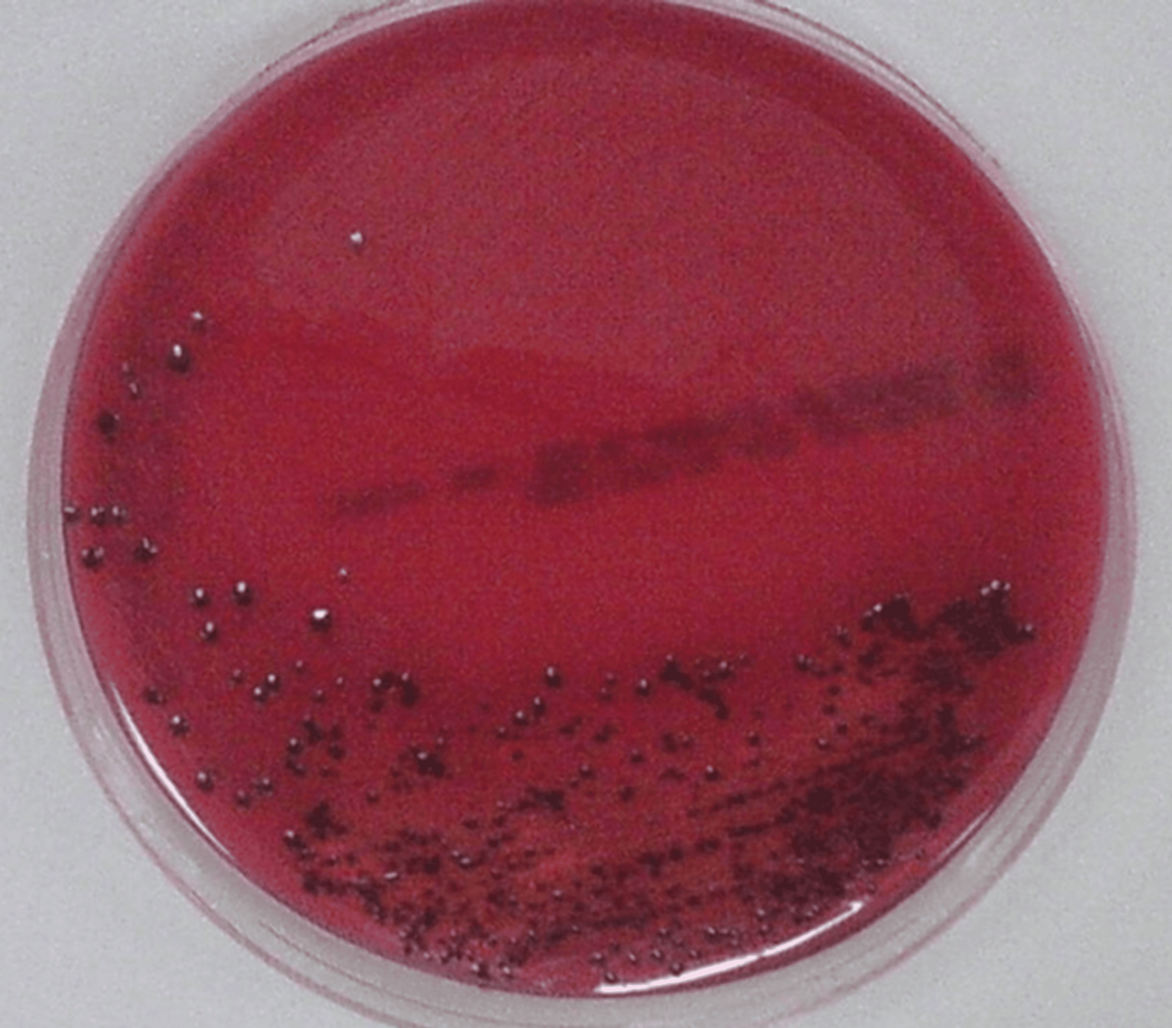
Colonization of Porphyromonas gingivalis showing black colonies in blood agar

**Figure 9:**
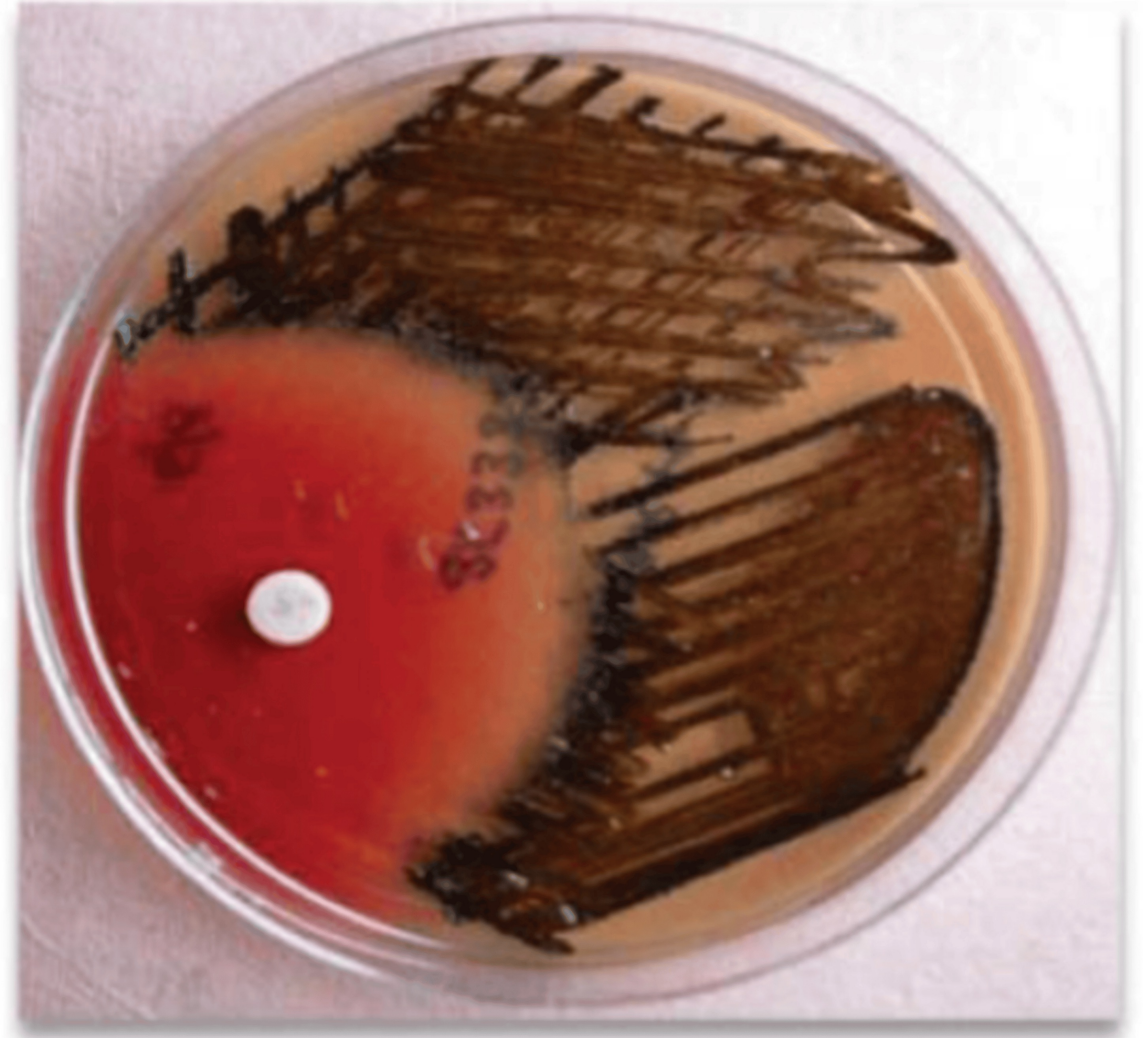
Black pigmented colonies of Porphyromonas gingivalis with metronidazole sensitivity

Identification of microorganisms was performed by the investigator under the supervision of the microbiologist and laboratory technician. The root canal samples in RTF were inoculated into boiled, cooled Robertson cooked meat medium and subcultured on laked blood agar and neomycin blood agar. The samples were incubated at 37°C anaerobically in the anaerobic BD GasPak system (Becton, Dickinson and Company, Franklin Lakes, NJ) for seven days. Identification of *P. gingivalis *was done as per standard procedure using biochemical tests and microscopic analysis. Biochemical identification of *P. gingivalis* included growth characteristics on blood agar, Gram stain morphology, catalase, lipase, indole, urease, mobility, and glucose fermentation tests [16]. For the presumptive identification of anaerobes, a metronidazole disc of 5 μg was placed in the plates during incubation and examined for the characteristic black colonies of Porphyromonas species with a zone of inhibition to Metronidazole (Figures 8, 9).

Statistical analysis

Data analysis was performed using Statistical Package for the Social Sciences version 22.0 (IBM Corp., Armonk, NY). Normality of continuous variables was assessed with the Shapiro-Wilk test. Nonnormally distributed continuous variables, such as pocket depth and attachment loss, were compared between groups using the Mann-Whitney U test. Categorical variables, including gender, systemic condition, clinical signs, periodontal status, PAI score, and lesion size, were analyzed using the chi-square test or Fisher's exact test when expected counts were <5. A *P* value of <0.05 was considered statistically significant.

## Results

Microbiological findings

The present study investigated the root canal microbiota for the presence of *P. gingivalis* in 32 patients with endodontic-periodontal lesions using culture. *P. gingivalis* was positive in 46.9% of cases (15/32) (Table 1).

**Table 1:** Presence of P. gingivalis by culture in patients with endodontic-periodontal lesion

Test organism	Presence/absence	Frequency	Percentage
P. gingivalis	Negative	17	53.1
	Positive	15	46.9

Demographic distribution

Participants were aged between 30 and 70 years, with a mean age of 43 years. The majority were in the 40-49 year age group (Table 2). Among *P. gingivalis*-positive cases, 47.4% were men and 46.2% were women. No significant association was observed between gender and the presence of *P. gingivalis* (*P* >0.05) (Table 3).

**Table 2:** Age-wise distribution of study participants with presence of P. gingivalis in endodontic-periodontal lesion

Test organism	Age groups	Frequency	Percentage
Presence of *P. gingivalis*	30-39	7	46.6
	40-49	8	53.3

**Table 3:** Gender-wise distribution of study participants in endodontic-periodontal lesion with presence of P. gingivalis

Gender	*P. gingivalis*, n (%)		Total, n (%)	Chi-square value	*P* value
	Negative	Positive			
Male	10 (52.6)	9 (47.4)	19 (100)	0.005	0.95 (XX)
Female	7 (53.8)	6 (46.2)	13 (100)		

Clinical parameters

Pain was significantly associated with the presence of *P. gingivalis* (*P* = 0.001). Other symptoms, including sinus tract, swelling, and discoloration, showed no significant associations (Table 4).

**Table 4:** Distribution of different signs and symptoms among study participants with presence P. gingivalis in endodontic-periodontal lesion ^#^Fisher’s exact test ^*^P < 0.05 statistically significant; *P* > 0.05 nonsignificant (XX)

Symptoms	Yes/no	*P. gingivalis*, n (%)		Total, n (%)	*P* value
		Negative	Positive		
Sinus tract	No	16 (55.2)	13 (44.8)	29 (100)	0.59 (XX)^#^
	Yes	1 (33.3)	2 (66.7)	3 (100)	
Pain	No	9 (100.0)	0 (0)	9 (100)	0.001^*,#^
	Yes	8 (34.8)	15 (65.2)	23 (100)	
Swelling	No	15 (60.0)	10 (40)	25 (100)	0.21 (XX)^#^
	Yes	2 (28.6)	5 (71.4)	7 (100)	
Tender on percussion	Yes	17 (53.1)	15 (46.9)	32 (100)	-
Discoloration	No	15 (55.6)	12 (44.4)	27 (100)	0.65 (XX)^#^
	Yes	2 (40)	3 (60)	5 (100)	

Periodontal findings

Mean pocket depth (5.3 mm) and attachment loss (4.93 mm) were higher in *P. gingivalis*-positive cases compared to negatives. Mann-Whitney U test showed a significant association between pocket depth and *P. gingivalis *(*P* = 0.005). Fisher's exact test revealed a significant association with furcation involvement (*P* = 0.02), but not with tooth mobility (*P *= 0.88) or bone loss (*P *= 0.73) (Tables 5, 6).

**Table 5:** Association of P. gingivalis with mean pocket depth and attachment loss ^*^P < 0.05 statistically significant; *P* > 0.05 nonsignificant (XX)

Presence of *P. gingivalis*		N	Mean (SD), mm	Range	Median	Interquartile range	Mann-Whitney U test	
							U statistic	*P* value
Pocket depth	Absent	17	3.59 (1.66)	2-7	3	2-5	53.5	0.005^*^
	Present	15	5.33 (1.35)	4-8	5	4-7		
Attachment loss	Absent	17	4.59 (3.59)	0-9	5	0-8	119.5	0.76 (XX)
	Present	15	4.93 (3.86)	0-10	6	0-8		

**Table 6:** Association of P. gingivalis with mobility, furcation involvement, PDL widening, and bone loss ^#^Fisher’s exact test ^*^P < 0.05 statistically significant; *P* > 0.05 nonsignificant (XX) PDL: periodontal ligament

Periodontal status	Score	*P. gingivalis*, n (%)		Total, n (%)	*P* value
		Negative	Positive		
Mobility	No	1 (33.3)	2 (66.7)	3 (100)	0.88 (XX)^#^
	Grade 1	10 (55.6)	8 (44.4)	18 (100)	
	Grade 2	6 (54.5)	5 (45.5)	11 (100)	
Furcation	No	14 (60.9)	9 (39.1)	23 (100)	0.02^*,#^
	Yes	1 (11.1)	8 (88.9)	9 (100)	
PDL widening	Yes	17 (53.1)	15 (46.9)	32 (100)	-
Bone loss	No	2 (100)	0 (0)	2 (100)	0.73 (XX)^#^
	Angular	4 (57.1)	3 (42.9)	7 (100)	
	Both	7 (53.8)	6 (46.2)	13 (100)	
	Horizontal	4 (44.4)	5 (55.6)	9 (100)	
	Vertical	0 (0)	1 (100)	1 (100)	

 Radiographic findings

Based on the criteria by Ørstavik et al., PAI scores of 3 or higher were considered. A PAI score of 5 was most frequent in *P. gingivalis-*positive cases (53%), with a significant association between this score and the presence of *P. gingivalis* (Table 7). A periapical lesion size of 3-5 mm was observed in 60% and >5 mm in 40% of *P. gingivalis*-positive cases, with a significant association between lesion size and *P. gingivalis* detection (*P* = 0.001) (Table 8).

**Table 7:** Comparison of PAI scores of study participants with presence of P. gingivalis ^#^Chi-square test ^*^P < 0.05 statistically significant; *P* > 0.05 nonsignificant (XX) PAI: periapical index

PAI score	Score	*P. gingivalis*, n (%)		Total, n (%)	Chi-square value	*P* value
		Negative	Positive			
	3	4 (23.5)	3 (20)	7 (21.9)	0.01	0.001^*,#^
	4	7 (41.2)	4 (26.7)	11 (34.4)		
	5	6 (35.3)	8 (53.3)	14 (43.8)		

**Table 8:** Comparison of periapical lesion size of study participants with presence of P. gingivalis ^#^Chi-square test ^*^P < 0.05 statistically significant; *P* > 0.05 nonsignificant (XX)

Periapical lesion size	Score	*P. gingivalis*, n (%)		Total, n (%)	Chi-square value	*P* value
		Negative	Positive			
	3-5 mm	17 (100)	9 (60)	26 (81)	0.01	0.001^*,#^
	>5 mm	0 (0)	6 (40)	6 (19)		

## Discussion

The microbial profile of the periodontal pocket and root canal in teeth affected by endodontic-periodontal lesions is largely similar, predominantly comprising anaerobic organisms. These lesions are characteristically polymicrobial, with facultative anaerobes such as* P. gingivalis, Prevotella intermedia,* and Fusobacterium commonly dominating the bacterial communities [4,5,16,17]. *P. gingivalis *is a black-pigmented, Gram-negative, nonfermenting obligate anaerobe that primarily inhabits the human oral cavity. Recognized as a major periodontopathogen, it is frequently isolated from both primary and secondary endodontic-periodontal infections, supporting its key role in the pathogenesis of these lesions [16].

Despite the limitations inherent to microbial culturing techniques, such as underestimation of certain species due to growth requirements, culture remains a conventional standard procedure in endodontic microbiology [17]. In the current study, *P. gingivalis* was isolated by culture methods in 46.9% of the cases. In comparison, Jacinto et al. identified the organism in 28% of cases with mixed endodontic infections [18]. Gomes et al. utilized polymerase chain reaction (PCR) to detect *P. gingivalis* in root-filled teeth with periapical lesions and reported a prevalence of 35.6% [19]. Mättö et al. observed a higher detection rate of 66% in saliva samples from individuals aged 31-80 years using PCR [20]. Conversely, Siqueira et al. reported only 4% detection in the apical third of infected root canals [21]. The greater detection rate observed in the present study may reflect differences in sampling methods, lesion types, or microbiological techniques used across studies.

In the present study, the highest prevalence of *P. gingivalis* was noted in individuals aged 40-49 years. Among these, 47.4% were men and 46.2% were women. This slight difference contrasts with the findings of Alwan, who reported a higher prevalence in men [22]. The minimal gender variation observed in our study could be attributed to the relatively small sample size. The mean age of affected individuals was 43 years, which aligns closely with the findings of Didilescu et al., who reported a mean age of 46 years among *P. gingivalis-*positive patients [6]. These findings suggest that *P. gingivalis *is not restricted to older populations and may also be prevalent among younger adults with endodontic-periodontal lesions.

*P. gingivalis* is often linked to clinical symptoms in endodontic-periodontal infections. In the current study, its presence showed a statistically significant association with symptomatic cases, particularly pain. Among the *P. gingivalis-*positive subjects, swelling was observed in 71.4%, and a sinus tract was present in 66.7% of cases. The formation of a sinus tract typically reflects the chronic nature of the lesion, as purulent exudate from necrotic pulp travels through the path of least resistance. Similar associations between *P. gingivalis* and signs such as swelling and sinus tract formation have been reported in previous culture-based studies [21,23]. Siqueira and Rôças detected *P. gingivalis* in 30% of symptomatic primary root canal infections [17], while Jacinto et al. found links between Porphyromonas spp. and symptoms such as tenderness to percussion, swelling, and moist canals [18].

The pathogenicity of *P. gingivalis* is largely attributed to its virulence factors, including a capsule and LPS, which are surface-exposed and contribute to antigenicity, hemagglutination, and adhesion. Additionally, its cell-bound collagenolytic enzymes enhance tissue degradation, reinforcing its role as a key pathogen in endodontic-periodontal lesions [18].

A comprehensive periodontal examination in the present study revealed a mean probing depth of 5.33 mm and clinical attachment loss of 4.93 mm among *P. gingivalis-*positive cases. These findings suggest a strong association between the presence of this pathogen and periodontal breakdown. A statistically significant association was observed between the presence of *P. gingivalis* and furcation involvement (*P* = 0.02) in patients with endodontic-periodontal lesions, suggesting a potential role of this pathogen in the progression of periodontal destruction in anatomically complex areas. However, no significant association was found between the presence of *P. gingivalis* and mobility (*P* = 0.88) or bone loss (*P* = 0.73). Previous studies by Gomes et al. have demonstrated similar associations between Porphyromonas species and periodontal parameters such as clinical attachment loss and percussion tenderness. The invasion of epithelial cells by *P. gingivalis* initiates host immune responses and inflammatory cascades, which ultimately result in soft and hard tissue destruction, manifesting as increased probing depths and attachment loss [17,18].

Endodontic-periodontal lesions in the present study were assessed using the PAI scoring system, based on the criteria by Ørstavik et al., which has been validated in several studies [14]. Only nonvital teeth with a PAI score of ≥3 were included. Notably, a PAI score of 5, indicative of severe periapical pathology, was observed in 53% of *P. gingivalis-*positive cases. These high scores reflect advanced bone demineralization and radiographic evidence of radiolucency with radiating alterations in bone structure.

This observation aligns with findings by Sundqvist et al., who reported a correlation between *P. gingivalis* and both asymptomatic periapical destruction and acute periapical inflammation [24]. Similarly, Wilson et al. demonstrated a significant association between *P. gingivalis* and periapical bone loss. In such cases, bone destruction is believed to result from antigenic stimulation originating in the root canal. Surface-associated lipopolysaccharide (LPS), free components of *P. gingivalis*, including capsular material, S-layer proteins, extracellular slime, and fibrils, have been shown to inhibit osteogenesis and promote bone resorption ​​​​​​​[25]​​​​​​​. These findings are further supported by both human and animal studies, which have demonstrated a higher prevalence of infection and increased lesion size in the presence of *P. gingivalis *[17,18,25].

The findings of this study emphasize the role of *P. gingivalis* in aggravating clinical signs such as pain, pocket depth, furcation involvement, and radiographic severity of endodontic-periodontal lesions. Early detection of this pathogen may help clinicians anticipate more severe disease presentations and tailor treatment strategies accordingly. Adjunctive antimicrobial therapy or host-modulatory approaches may be considered in cases with confirmed *P. gingivalis* involvement to improve clinical outcomes and prevent progression of endodontic-periodontal lesions.

One limitation of the present study was the relatively small sample size and its focus on a single microorganism, *P. gingivalis*. As endodontic-periodontal lesions are typically polymicrobial, a broader microbial analysis is warranted. Future studies should incorporate larger cohorts and evaluate a wider range of microbial species to better understand the correlations between clinical signs and the complex microbial ecology of necrotic root canals.

## Conclusions

Within the limitations of this study, it can be concluded that *P. gingivalis* was significantly associated with clinical signs and symptoms of endodontic-periodontal lesions, particularly pain. Its presence was more frequent in symptomatic cases and correlated with increased lesion severity. Cases positive for *P. gingivalis* demonstrated a higher prevalence of infection and larger lesion sizes, along with advanced radiographic features such as bone demineralization and structural changes. These findings highlight the pathogenic role of *P. gingivalis* in the progression of endodontic-periodontal disease. Future in vitro and clinical studies with larger sample sizes and broader microbial profiling are essential to better understand the relationship between specific pathogens and the clinical manifestations of these complex lesions.

## References

[1] Al-Sibassi A, Niazi SA, Clarke P, Adeyemi A (2025). Management of the endodontic-periodontal lesion. Br Dent J.

[2] Chen B, Zhu Y, Lin M (2024). Expert consensus on the diagnosis and therapy of endo-periodontal lesions. Int J Oral Sci.

[3] Evans M (2023). The endodontic-periodontal juncture: where two worlds meet. An overview of endo-perio lesions. Aust Dent J.

[4] Das AC, Sahoo SK, Parihar AS, Bhardwaj SS, Babaji P, Varghese JG (2020). Evaluation of role of periodontal pathogens in endodontic periodontal diseases. J Family Med Prim Care.

[5] Lopes EM, Passini MR, Kishi LT, Chen T, Paster BJ, Gomes BP (2021). Interrelationship between the microbial communities of the root canals and periodontal pockets in combined endodontic-periodontal diseases. Microorganisms.

[6] Didilescu AC, Rusu D, Anghel A (2012). Investigation of six selected bacterial species in endo-periodontal lesions. Int Endod J.

[7] von Elm E, Altman DG, Egger M, Pocock SJ, Gøtzsche PC, Vandenbroucke JP; STROBE Initiative (2007). The Strengthening the Reporting of Observational Studies in Epidemiology (STROBE) statement: guidelines for reporting observational studies. Lancet.

[8] Patro S, Meto A, Mohanty A (2022). Diagnostic accuracy of pulp vitality tests and pulp sensibility tests for assessing pulpal health in permanent teeth: a systematic review and meta-analysis. Int J Environ Res Public Health.

[9] Al Shayeb KN, Turner W, Gillam DG (2014). Periodontal probing: a review. Prim Dent J.

[10] Barbosa VL, Angst PD, Finger Stadler A, Oppermann RV, Gomes SC (2016). Clinical attachment loss: estimation by direct and indirect methods. Int Dent J.

[11] Lang NP, Joss A, Orsanic T, Gusberti FA, Siegrist BE (1986). Bleeding on probing. A predictor for the progression of periodontal disease?. J Clin Periodontol.

[12] Varadhan KB, Parween S, Bhavsar AK, Kumawat RK, Ramchandran S, Varma S (2019). Tooth mobility measurements: realities and limitations. J Evol Med Dent Sci.

[13] Pilloni A, Rojas MA (2018). Furcation involvement classification: a comprehensive review and a new system proposal. Dent J (Basel).

[14] Ørstavik D, Kerekes K, Eriksen HM (1986). The periapical index: a scoring system for radiographic assessment of apical periodontitis. Endod Dent Traumatol.

[15] Miletić I, Jukić S, Anić I, Zeljezić D, Garaj-Vrhovac V, Osmak M (2003). Examination of cytotoxicity and mutagenicity of AH26 and AH Plus sealers. Int Endod J.

[16] Pereira R, Arboleda S (2020). A multidisciplinary approach of an endo-perio lesion in a severely compromised tooth: an 18-year follow-up case report. J Med Life.

[17] Siqueira JF Jr, Rôças IN (2005). Exploiting molecular methods to explore endodontic infections: part 2--redefining the endodontic microbiota. J Endod.

[18] Jacinto RC, Gomes BP, Shah HN, Ferraz CC, Zaia AA, Souza-Filho FJ (2006). Incidence and antimicrobial susceptibility of Porphyromonas gingivalis isolated from mixed endodontic infections. Int Endod J.

[19] Gomes BP, Pinheiro ET, Jacinto RC, Zaia AA, Ferraz CC, Souza-Filho FJ (2008). Microbial analysis of canals of root-filled teeth with periapical lesions using polymerase chain reaction. J Endod.

[20] Mättö J, Saarela M, Alaluusua S, Oja V, Jousimies-Somer H, Asikainen S (1998). Detection of Porphyromonas gingivalis from saliva by PCR by using a simple sample-processing method. J Clin Microbiol.

[21] Siqueira JF Jr, Rôças IN, Alves FR, Santos KR (2004). Selected endodontic pathogens in the apical third of infected root canals: a molecular investigation. J Endod.

[22] Alwan AH (2024). The impact of age and gender on periodontal conditions in Iraqi people: a retrospective study. Al‑Rafidain J Med Sci.

[23] Siqueira JF Jr, Rôças IN (2022). Present status and future directions: microbiology of endodontic infections. Int Endod J.

[24] Sundqvist G, Johansson E, Sjögren U (1989). Prevalence of black-pigmented Bacteroides species in root canal infections. J Endod.

[25] Wilson M, Reddi K, Henderson B (1996). Cytokine-inducing components of periodontopathogenic bacteria. J Periodontal Res.

